# A Platform to Record Patient Events During Physiological Monitoring With Wearable Sensors: Proof-of-Concept Study

**DOI:** 10.2196/10336

**Published:** 2019-01-03

**Authors:** Jonathan Duc Vinh Vo, Alexander M Gorbach

**Affiliations:** 1 Infrared Imaging and Thermometry Unit National Institute of Biomedical Imaging and Bioengineering National Institutes of Health Bethesda, MD United States

**Keywords:** data collection, mobile phone, mobile-based platform, patient journal, sensors

## Abstract

**Background:**

Patient journals have been used as valuable resources in clinical studies. However, the full potential value of such journals can be undermined by inefficiencies and ambiguities associated with handwritten patient reports. The increasing number of mobile phones and mobile-based health care approaches presents an opportunity to improve communications from patients to clinicians and clinical researchers through the use of digital patient journals.

**Objective:**

The objective of this project was to develop a smartphone-based platform that would enable patients to record events and symptoms on the same timeline as clinical data collected by wearable sensors.

**Methods:**

This platform consists of two major components: a smartphone for patients to record their journals and wireless sensors for clinical data collection. The clinical data and patient records are then exported to a clinical researcher interface, and the data and journal are processed and combined into a single time-series graph for analysis. This paper gives a block diagram of the platform’s principal components and compares its features to those of other methods but does not explicitly discuss the process of design or development of the system.

**Results:**

As a proof of concept, body temperature data were obtained in a 4-hour span from a 22-year-old male, during which the subject simultaneously recorded relevant activities and events using the iPhone platform. After export to a clinical researcher’s desktop, the digital records and temperature data were processed and fused into a single time-series graph. The events were filtered based on specific keywords to facilitate data analysis.

**Conclusions:**

We have developed a user-friendly patient journal platform, based on widely available smartphone technology, that gives clinicians and researchers a simple method to track and analyze patient activities and record the activities on a shared timeline with clinical data from wearable devices.

## Introduction

There have been an increasing number of telehealth and mobile device health care management approaches based on the emerging field of patient-centered outcomes, which focuses on medical outcomes related to metrics of physiology and quality of life. For example, one modern care management approach used an electronic health record system as a child physical abuse alert system [1]. Additionally, care management has been implemented with information technology to improve asthma outcomes in adult populations and mobile apps have been used to encourage compliance with oral chemotherapy and symptom management [2,3]. The Institute of Medicine has reported that improvements in communication between patients and clinicians are needed to reduce health inequalities, and these improvements have begun to focus on telehealth and the use of mobile devices. One example of such an improvement is the use of mobile devices in sleep medicine [4,5]. One possibility to bridge the gap in communication between patients and clinicians is through the use of mobile-based digital records that function as journals for patients to record their events and activities securely and remotely. These have potential uses in clinical research, as well as in clinical practice.

Currently, handwritten patient-recorded events are typically incomplete or used inefficiently due to ambiguity about what should be reported, absence of standard vocabulary to describe events, the inconvenience of reporting events at the times they occur, inaccurate timing, and difficulties with reading patients’ handwriting and aligning the records with clinical physiological data. However, there is limited peer-review science comparing handwritten patient journals to electronic journals.

Moreover, although there are current options for health and symptom-logging apps on smartphones, these apps lack the ability to use voice dictation for hands-free recording, preset graphical images to facilitate use by English as a second language (ESL) or nonliterate patients, data export, integration of events with physiology data, or data analysis features [6-9].

To collect physiological data and patient reports on the same timeline on a single mobile device, we propose a proof of concept to simplify patient reporting by using iMessage on a dedicated iPhone and to combine and align clinical data with patient-reported data in a unified digital journal. The aim and expectation, pending confirmation in future clinical trials, is that the smartphone’s ability to collect physiological data wirelessly and to have multiple options for recording patient events will allow for increased ease of use for patients and for more accurate characterization of clinically valuable events, as determined by researchers, during long-term monitoring by reducing barriers that limit the accuracy of the patient record such as limited proficiency in English or limited motor function in the extremities.

Continuous temperature monitoring with skin temperature patches has been performed by a previous group [10] that focused on using wearable electronics or cell phones. Our approach for this proof of concept was to use iMessage software on iPhone in conjunction with temperature patches to combine skin temperature data with patient reports of events and symptoms on the same timeline.

## Methods

### Concept and Block Diagram

**Figure 1:**
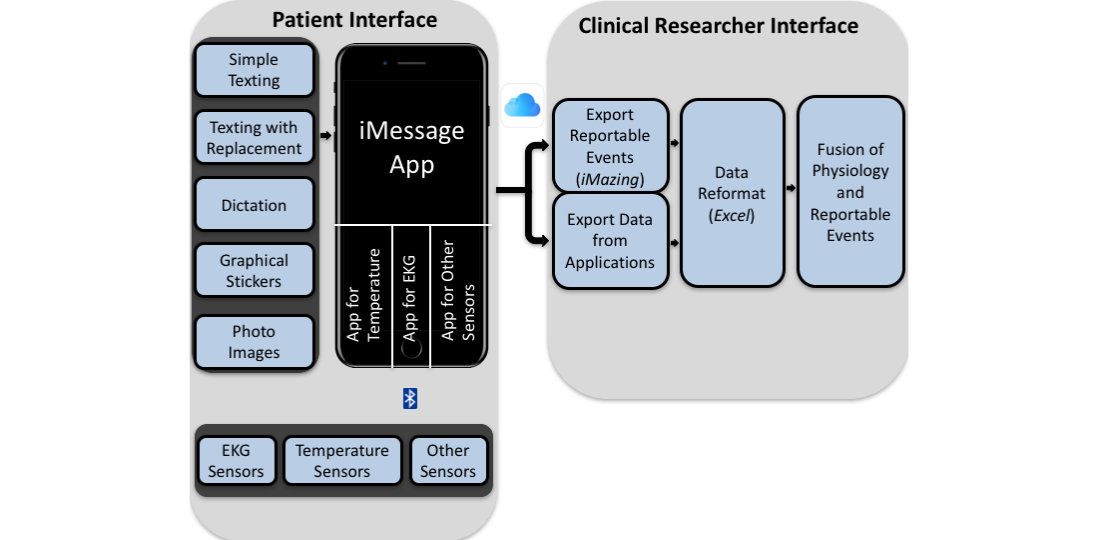
Block diagram depicting the fusion of physiological data and patient-reportable events data into one time-series graph. EKG: electrocardiogram.

We implemented the concept of using a secure iPhone, along with its various capabilities in iMessage, for clinical research purposes, as seen in Figure 1.

The Patient Interface consists of body sensors (electrocardiogram, EKG; temperature; etc) and a dedicated iPhone wirelessly connected via Bluetooth to the sensors. iPhone capability includes the built-in iMessage app, which allows simple texting; texting with replacements of abbreviations, which can be used to implement a preset vocabulary for specific clinical research; dictation (voice recognition); graphical stickers, which are preset graphical images or icons designed for specific clinical research, for example, monitoring of heart fibrillation with EKG, circadian temperature changes, and pain scores in time and location; and photo images collected by the patient.

The Clinical Research Interface consists of a Mac Pro desktop (2013, Apple Inc), a lightning cable to export reportable events, and a portable flash drive (c20i JumpDrive, Lexar) to export physiological data from apps (in future versions of the platform, the flash drive can be replaced with a more secure cloud solution that is encrypted in both directions). The desktop software includes iMazing (2.5.3, DigiDNA) to export reportable events to an Excel (15.34, Microsoft) format and MATLAB (R2017a, Mathworks).

First, a dedicated iPhone will be given to each patient. Patients will then text, dictate, use graphical stickers, or collect photo images in iMessage to report events predesignated by clinicians. Physiological sensors will be placed on the patients’ bodies. Applications will be activated, and patients may then go to their hospital room or possibly to their home. The duration of monitoring is dependent on acquisition frequency and the battery capacity of the iPhone and sensors. Second, at the end of the monitoring period, patients will send the iPhone back to the clinical facility where the clinical researcher will download the reportable events by connecting the iPhone and Apple desktop via the lightning cable, and after that, the researcher will export physiological data via a portable flash drive. Third, data will be reformatted on the desktop through Excel, which allows them to be readable by a MATLAB script. Finally, the MATLAB script will generate a graph that fuses the physiological data and reportable events, allowing visualization of physiological changes and subsequent reportable events on the same time scale. These data can be filtered based on specific reportable events, duration of the events, or any other clinical research criteria.

### Hardware

For the Patient Interface, we used an iPhone (version 6, Apple Inc), though more recent versions should work as well, and a HomePod (2017 version, Apple Inc), which acts as a microphone for patients to use Siri dictation. For temperature monitoring demonstration purposes, we used the Temp Pal skin temperature sensor (model STP-MB01-1, iWEECARE Co) [9]. On the Clinical Researcher Interface, we used a Mac Pro (2013, Apple Inc), a Lightning-to-USB cable (Apple Inc), and a portable flash drive (model c20i JumpDrive, Lexar Inc).

We implemented the temperature acquisition process using wearable skin temperature patches connected via Bluetooth to an iPhone, which allowed us to also create patient reports and combine them with physiological data on a dedicated iPhone device.

### Software

**Figure 2:**
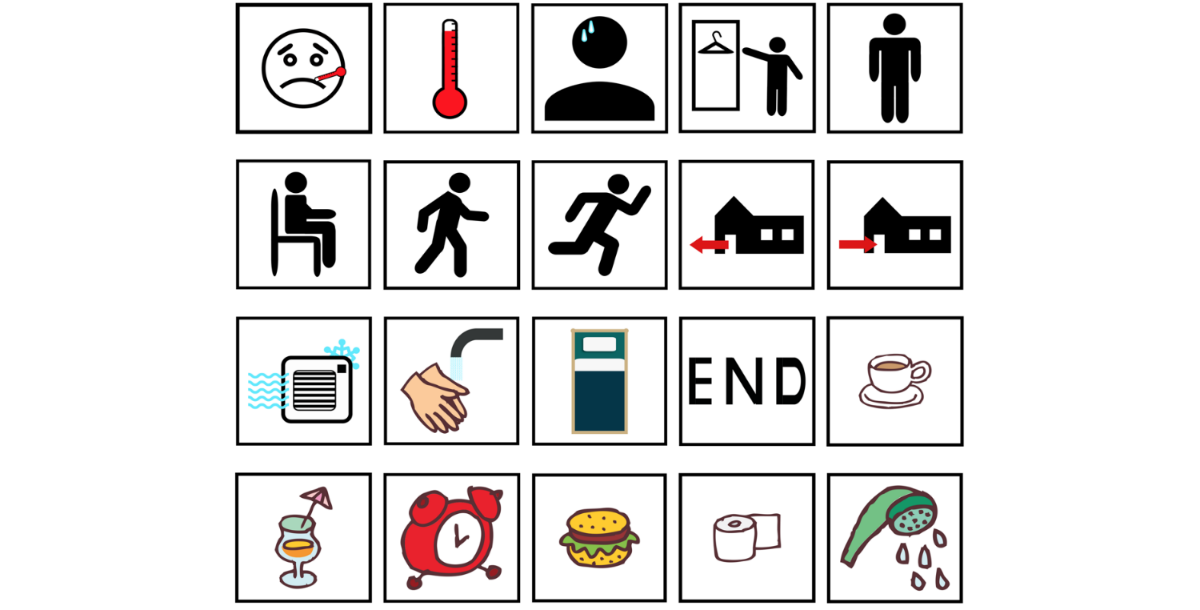
Twenty stickers preset in Assembly for use in patient journals.

There are several iPhone capabilities that we used to create the patient reports. First, with Apple’s intelligent personal assistant, Siri, it is possible to use voice recognition so that patients will be able to dictate their records. Second, Siri can be used in a hands-free manner by speaking the keywords “Hey Siri.” Next, with Apple’s instant messaging service, iMessage, patients can securely create time-stamped logs of their events and activities. iMessage has the capability of using both texting and voice dictation. With either method, patients send themselves a text message containing what they wish to record, and the text message serves as a digital log of their activities and events. Third, iPhones come with a text replacement feature for typing, whereby users can set keywords or letters that will then autocomplete to form predefined words, phrases, or sentences. This will allow patients to type their records easily and accurately (with fewer keystrokes and fewer typing mistakes or misspellings) while also avoiding slightly different written versions of the same type of event. This will also give patients a standardized dictionary for specific keywords and events. Finally, iMessage allows for the use of “stickers” in addition to text, so patients can generate messages visually and quickly from a standardized dictionary preset by the clinical researchers. Using the iPhone app *Assembly* (version 1.5.7, Pixite LLC), which allows for the creation and customization of stickers, we have a set of 20 stickers that patients can choose from to use in their journals (Figure 2) [11]. The stickers are converted to text using MATLAB (R2017a). The stickers in Figure 2 respectively represent: “Fever,” “Heating,” “Sweating,” “Changing Clothes,” “Standing,” “Sitting,” “Walking,” “Running,” “Outside,” “Inside,” “Air Conditioner,” “Washing Hands,” “Sleeping,” “End,” “Hot Drink,” “Cool Drink,” “Waking Up,” “Eating,” “Bathroom,” and “Shower.”

Along with iMessage, we used the *TempPal* app (version 1.5.1, iWEECARE Co) to record skin temperature simultaneously with the reportable events [11]. Finally, we used the *MobileManager* app (1.2.0, Lexar Inc) on iPhone to transfer temperature data from iPhone to Mac.

On Mac, we used the third-party app *iMazing* (version 2.5.3, DigiDNA Sàrl Inc) to export iMessage texts into a.csv format [12]. *iMazing* is a simple and user-friendly back-up and transfer or export app for iPhone. We used *Excel* (version 15.34, Microsoft Co) to reformat the temperature data and reportable events data. Finally, using MATLAB (version R2017a, MathWorks Inc), we created a simple script to extract data from both files and combine and align the temperature data with patient-recorded events into one single graph. If patients used stickers in their journals, the stickers are converted to text in the script as well. Multimedia Appendix 1 presents a more in-depth operating procedure.

## Results

**Figure 3:**
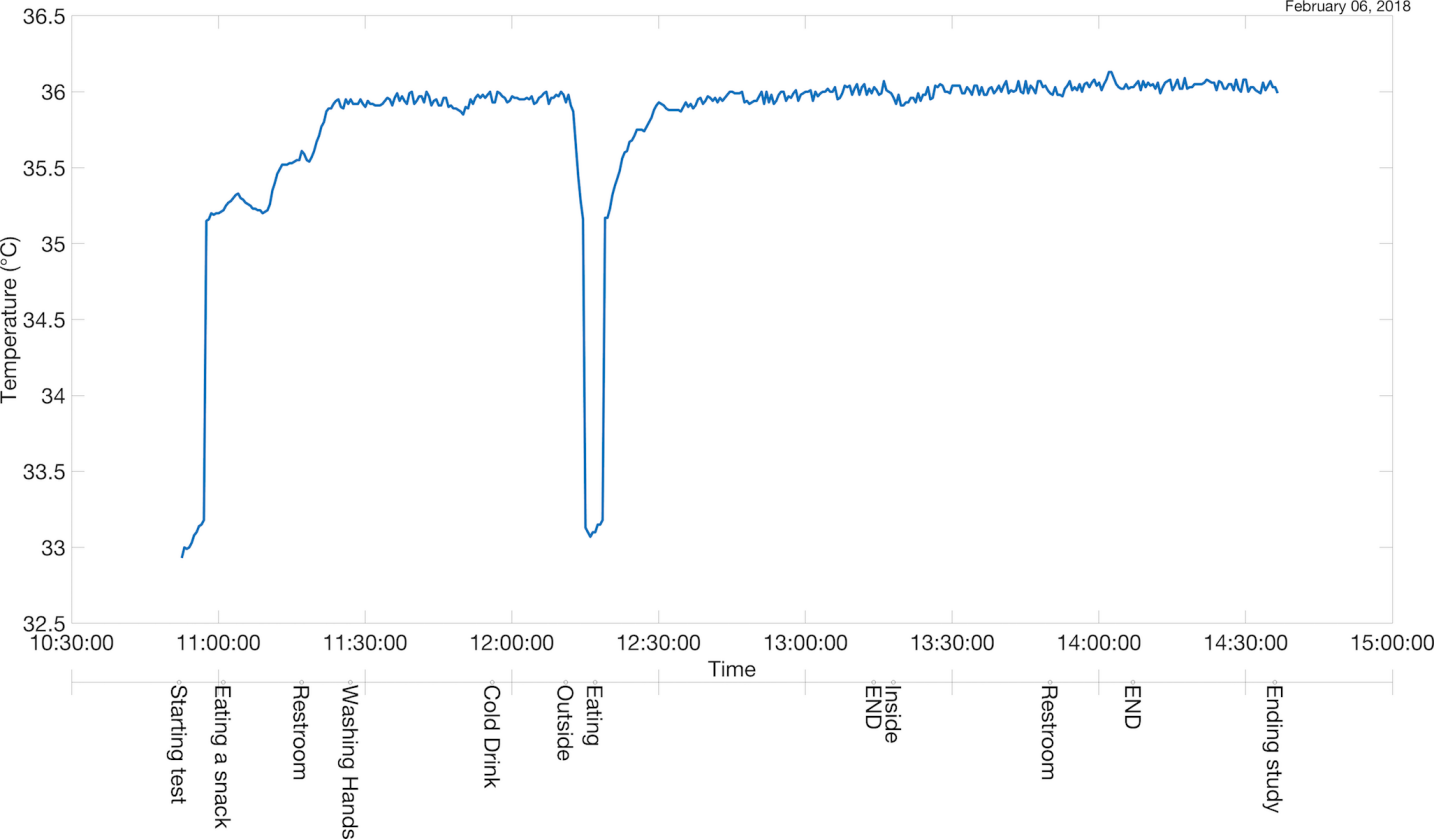
Temperature data taken for 4 hours of using a TempPal temperature sensor.

As a simple test of our prototype, a healthy 22-year-old male used the platform described here to record his activities and events over a 4-hour session, while a sensor recorded his body temperature. The data were processed on a Mac Pro desktop and combined into a single time-series graph, an example of which is shown in Figure 3. The sensor was placed on the right chest of a healthy 22-year-old male. The patient logged activities and events using iMessage text-typing and Siri. Below the time axis is the dictation axis, which shows the patient-recorded notes at each corresponding time point. The words “END” denote that the prior event or activity has been completed.

**Figure 4:**
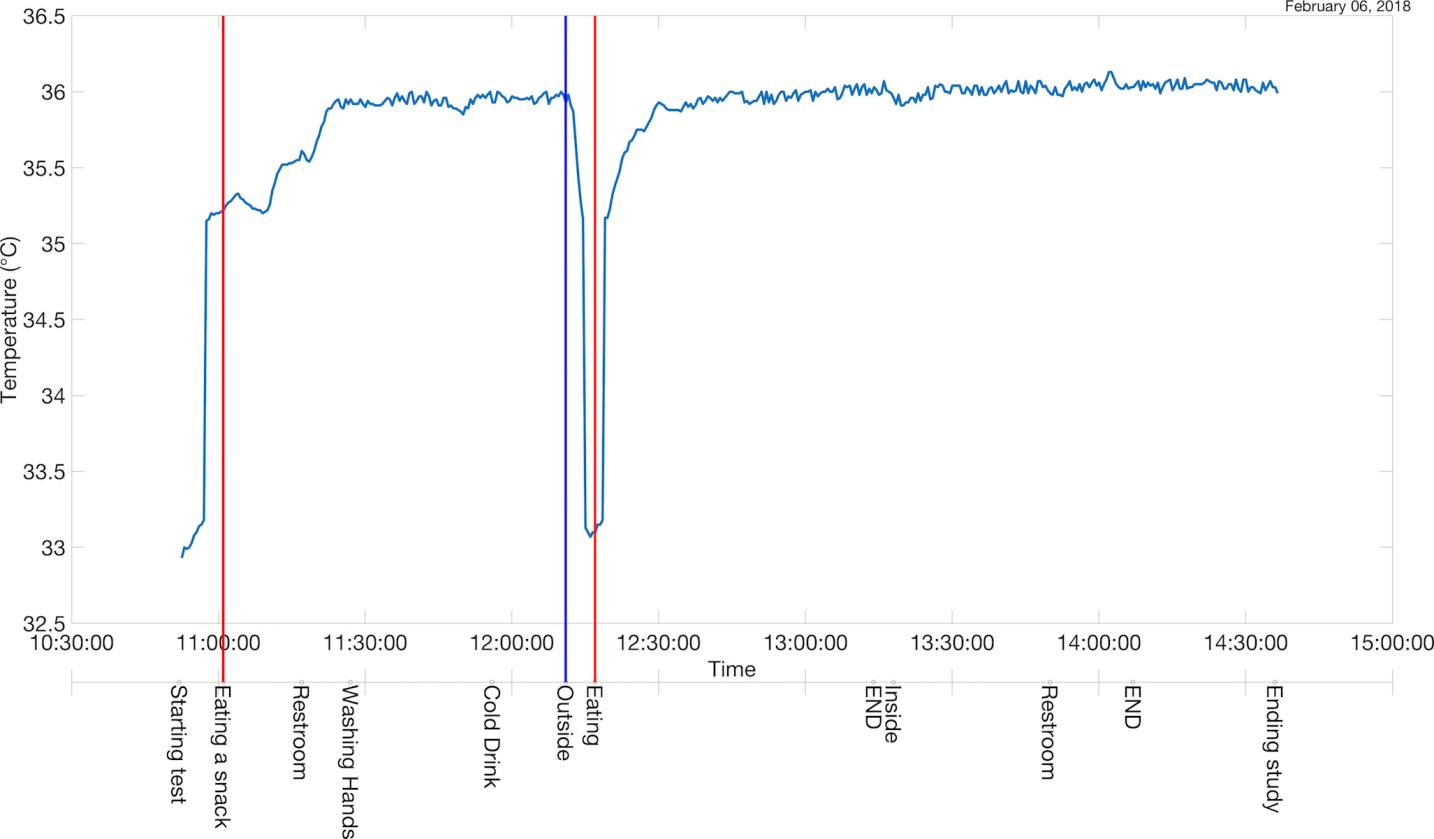
Filtering of the patient journal using two keywords: “eating” and “outside”.

The journal was filtered using the keywords “eating” and “outside,” resulting in a time-series graph (Figure 4) highlighting the times at which the subject logged any activities with the words “eating” or “outside.” The words “END” denote that the prior event or activity has been completed. The blue vertical line denotes the point when the patient was outside, and the red lines denote the points when the patient is eating. The variable blue line shows a temperature decrease as the patient walks outside, which matches the drop in temperature moving from a heated room to outside the building.

Using the filtered time-series graph, we can easily see that when the subject moved outside, his body temperature decreased, and when he began eating, his body temperature began to increase. Although the patient only recorded his daily activities while temperature was measured, in a more clinically relevant study, other physiological metrics, such as cardiac data, might be used.

## Discussion

### Principal Findings

We have designed and tested a prototype capable of recording a patient’s comments synchronously with physiological data (such as temperature, as used in this paper) from wearable devices. A shared timeline for comments and data is essential for accurate interpretation of data that can be influenced by the patient’s activities (exercising, eating, showering, etc) or mood. Our prototype served as a proof of concept and demonstrated that this can be accomplished, without the need for continuous involvement of medical personnel. Voice dictation and graphical “stickers” are offered as alternatives to texting, opening the availability of the method to a wide population of patients, including ESL and nonliterate patients or those without the ability to type. The particular texting system used in our prototype (iMessage) is extremely secure due to end-to-end encryption. The method is also able to combine patient comments (journals) and physiological data into a single graph and filter the journal entries with specific keywords for ease of data analysis.

Our method may also be applied to the field of predictive medicine, which aims to identify patients who are at risk of disease and thereby facilitating steps to treat the disease early or even to prevent it. These goals benefit from accurate, patient-recorded events combined and aligned with physiological data. Although we chose to focus on temperature monitoring in this demonstration, our mobile-based platform is, of course, usable with other sensors. For example, patients can undergo remote cardiac monitoring using either wearable devices that send data to medical personnel or devices that could contain automated detection algorithms themselves [13,14]. Because our platform is already mobile, switching to cardiac monitoring from temperature monitoring would just involve switching the temperature sensors to EKG sensors. Unlike traditional cardiac monitoring sessions, however, our platform does not use handwritten diaries [15].

### Limitations

There are some drawbacks to our methods. The prototype currently relies on having a connection to either Wi-Fi or cellular data networks, which may be difficult in some settings. One of the configurations for the future could be a system that is locally based on the device (ie, does not require a constant network connection). Such versions could record all patient event data locally and automatically synchronize the data with the cloud the next time that the device has network access, which would also remove the need for a portable flash drive. This is a common technical problem that has been solved in other mobile apps where there is no continuous network access.

Additionally, because of the secure end-to-end encryption of Apple’s iMessaging app, our prototype currently focuses on iPhone and Apple systems exclusively and not on Android phones. This, however, is a relatively minor drawback as the basic principles for our proof of concept can be easily transferable to Android phones.

We have also not evaluated the usability or user friendliness of our platform with patients, researchers, or clinicians at this time; however, we hope to test our platform in the future in clinical studies.

Finally, although the use of a portable USB drive for data transfer between iPhone and Mac desktop may prove more cumbersome than wireless or cloud-based transfer methods, this was done to ensure the physical security of our data.

**Table 1:** Features of current approaches to monitor patient-recorded events.

Apps	Journal options	Integration with physiology	Analysis features
Diary Health App	Voice dictation; text with autocomplete	Yes	No
Symple App	Text with autocomplete	No	No
Penzu Health Diary	Text with autocomplete; photo images	No	No

Furthermore, in future iterations, the portable USB drive will be replaced with a cloud solution that is secured by encryption.

### Comparison With Prior Work

The *Diary Health App* (version 2.4.8, The Diary Co) allows for voice dictation and text options for patient-recorded events, and the app has some integration with basic physiological measures, such as heart rate and body temperature; however, the app does not allow for combining and aligning of the patient-recorded events and physiological data. It also does not offer user-friendly options of preset graphical Sticker images or photos in the patient journal [6].

The *Symple App* (version 2.1.9, Symple Health Inc) allows for text options for patient-recorded events; however, there is no integration with physiological data, and there are no options for preset stickers or photos [7].

The *Penzu Health Diary* (version 3.4.2, Penzu Inc) app allows for text and photo images for the patient-recorded events; however, there are no options for preset stickers, voice dictation, or integration with physiological data [8].

These apps also do not contain any data analysis or graph-generating features. Table 1 shows the **f**eatures of current approaches to monitor patient-recorded events. None of the approaches allow patients to use graphical stickers.

### Conclusion

The current standard of care (written journaling by the patient during physiological data collection) can be inaccurate in timing, inconvenient for patients to use, and difficult for researchers to interpret due to poor handwriting or lack of alignment of the journal entries with physiological data. By taking advantage of a smartphone’s capabilities and third-party programs, the proposed method offers greater convenience for the patient through choices of data entry methods, enables accurate journal recording and data collection on a single, shared timeline, and eliminates the common illegibility of written records. Other features, such as text filtering options also facilitate analysis.

This work is offered as a proof of concept. Future work could include evaluating the method’s performance in clinical trials that involve physiological monitoring. Additional developments could focus on reading iMessages on Mac directly from the iMessage database. This would remove the need to move the iPhone physically back to the medical facility for data transfer, though the iPhone and Mac would need to have the same dedicated iCloud account. Finally, a more ambitious goal may be to build a custom app and not use iMessage at all. This would allow researchers to have more control over the data format and input, for example allowing for only certain preset inputs rather than free-form texting. It would also allow for improved data management by having the data sent to a secure server or stored remotely via CloudKit. Furthermore, the app could run on the Apple watch, so that the data could be shared with family members and other members of the health care and research team. This would also give patients the ability to use their own iPhones and remove the need for special, dedicated iPhones. Additionally, the custom app would be able to include other iPhone features, such as the TrueDepth camera on iPhone X, which performs facial recognition. This could be developed with Apple’s publicly available face detection and recognition app program interface and used as part of a pain scale for patients.

## Appendix

Multimedia Appendix 1 Step-by-step operating procedure for platform.

## Abbreviations

EKG: electrocardiogram
ESL: English as a second language
