# Supplementary material for: A Platform to Record Patient Events During Physiological Monitoring With Wearable Sensors: Proof-of-Concept Study
Source: Interact J Med Res. 2019 Jan 3;8(1):e10336. doi: 10.2196/10336 (PMC6682266; doi:10.2196/10336)
Supplement: Multimedia Appendix 1 [file ijmr_v8i1e10336_app1.pdf]

Appendix 1

1. Pre-set vocabulary and graphical Stickers designed for the specific clinical research will be downloaded on the iPhone. Vocabulary for text replacement and graphical Stickers designed for the specific clinical research will be set-up on the dedicated iPhone. The vocabulary will be done by navigating to Text Replacement in the Keyboards settings of the iPhone. The graphical Stickers will be downloaded onto the iPhone using the *Assembly* application. Siri will be setup for the patient's voice dictation by navigating to the Siri & Search options in the iPhone settings.

2. Patients will then be given the dedicated iPhone. Body sensors wirelessly connected to the iPhone will be placed, and the app will be activated to begin data acquisition simultaneously while the patient begins to report their events and activities. To ensure appropriate use of the patient-reported events, patients will be taught the concept of Reportable Events and will be instructed to text (with replacement options), dictate, select appropriate Stickers, or collect photo images to report events in iMessage.

3. In order to use texting to record events, patients will open iMessage and send a text message to a contact named "Myself." This contact has the same phone number as the iPhone used to send the text message, so patients will be essentially sending a text message to themselves. In order to use text

replacement, patients will type the pre-set shortcut, which will then autocomplete into the pre-set phrase. To use Siri dictation, patients will have two options: a hands-free option and a hands-on option. For the iPhone 6 and older versions, the hands-free option can only be used if the iPhone is connected to a power supply. To use the hands-free option, patients will say the words “Hey Siri” and then tell Siri to send a text message to “Myself.” The patients will then dictate their message, and, after confirming that the message is correct, they will say “Send.” To allow for easier access to Siri, a HomePod (2017 version, Apple Inc.) will be placed in the same room as the patient. The HomePod will be linked to the dedicated iPhone, which will give patients the ability to use the hands-free Siri dictation option without a wired microphone and while speaking at normal volume in the room. To use the hands-on option, patients will navigate to the iMessage app and tap the dictation button in the text box, dictate their message, then send it. In order to use the pre-set graphical Sticker, patients will tap the App Drawer button, select the *Assembly* app, and choose the sticker to represent the event they are recording. Finally, to collect and send a photo image in iMessage, patients will tap the Camera button, take a photo of the image they wish to send, then send the photo.

4. Following the monitoring session, the iPhone will be sent back to the medical facility immediately so that the reportable events and physiological data may be transferred to the Mac Pro desktop via lightning cable and portable flash drive (model c20i JumpDrive, Lexar), respectively. After data reformatting in Excel, the

MATLAB script will read the data, convert the Stickers to text, and generate a graph that fuses the physiological data with the reportable events. For analysis, data will be filtered based on specific events, event duration, or their frequencies and temporal patterns.
